# Meta-Analysis of Incorporating Glucosinolates into Diets and Their Effects on Ruminant Performance, Ruminal Fermentation, Methane Emissions, Milk Composition, and Metabolic Biochemical Attributes

**DOI:** 10.3390/ani15101480

**Published:** 2025-05-20

**Authors:** Min Gao, Agung Irawan, Mohamed El-Sherbiny, Małgorzata Szumacher-Strabel, Adam Cieślak, Muhammad Ariana Setiawan, Hassan Jallal, Isa Fusaro, Anuraga Jayanegara, Yulianri Rizki Yanza, Yongbin Liu

**Affiliations:** 1 State Key Laboratory of Reproductive Regulation & Breeding of Grassland Livestock, Inner Mongolia University, Hohhot 010021, China; min.gao@imu.edu.cn; 2 National Sheep Genetic Evaluation Center, Inner Mongolia University, Hohhot 010070, China; 3 Vocational School, Universitas Sebelas Maret, Surakarta 57126, Indonesia; a.irawan@staff.uns.ac.id; 4 Department of Dairy Science, National Research Centre, 33 Bohouth St., Dokki, Giza 12622, Egypt; elsherbiny.nrc.eg@gmail.com; 5 Institute of Animal Nutrition, Nutrition Diseases and Dietetics, Faculty of Veterinary Medicine, University of Leipzig, An den Tierkliniken 9, 04103 Leipzig, Germany; 6 Department of Animal Nutrition, Poznań University of Life Sciences, Wołyńska 33, 60-637 Poznań, Poland; malgorzata.szumacher@up.poznan.pl (M.S.-S.); adam.cieslak@up.poznan.pl (A.C.); 7 Department of Animal Nutrition and Feed Technology, Faculty of Animal Husbandry, Universitas Padjadjaran, Sumedang 45363, Indonesia; muhammad20230@mail.unpad.ac.id; 8 Department of Veterinary Medicine, Faculty of Veterinary Medicine, University of Teramo, 64100 Teramo, Italy; hjalal@unite.it (H.J.); ifusaro@unite.it (I.F.); 9 Department of Animal Nutrition and Feed Technology, Faculty of Animal Science, IPB University, Bogor 16680, Indonesia; anuragaja@apps.ipb.ac.id; 10 Department of Animal Genetics, Breeding, and Reproduction, College of Animal Science, Inner Mongolia Agricultural University, Hohhot 010018, China

**Keywords:** *brassica*-derived feeds, enteric methane, glucosinolates, meta-analysis, ruminal fermentation

## Abstract

*Brassica*-derived feeds have been recognized for their economic and environmental benefits in ruminant nutrition. However, their utilization is constrained by the presence of glucosinolates and sulfur-containing compounds that exhibit both beneficial and adverse effects. This meta-analysis included 36 studies that evaluated the impact of glucosinolate intake on ruminant performance, nutrient digestibility, milk composition, and methane emissions. This analysis, conducted in accordance with PRISMA guidelines, revealed that glucosinolate supplementation resulted in a quadratic increase in milk urea nitrogen concentration (*p* = 0.017). Additionally, significant interactions between glucosinolate level and source influenced crude protein digestibility (*p* = 0.026). Milk composition parameters, including 4% fat-corrected milk, energy-corrected milk, milk protein, and lactose proportions, were significantly affected (*p* < 0.05). Furthermore, methane emissions (g/kg DMI) decreased quadratically with increasing glucosinolate intake (*p* = 0.003), with additional interactions observed between dietary treatments and animal species (*p* = 0.029). Propionate and isobutyrate concentrations increased in a quadratic and linear manner, respectively (*p* < 0.05). These findings suggest that glucosinolate-containing feed can enhance nutrient utilization and mitigate methane emissions in ruminants. However, the magnitude of these effects is dependent on the glucosinolate dosage, source, animal species, and dietary composition, necessitating further research to optimize their use in ruminant nutrition.

## Simple Summary

*Brassica*-derived feed has garnered attention as a cost-effective and sustainable source of protein for ruminants. However, its utilization is constrained by glucosinolates, which are naturally occurring compounds that exhibit both beneficial and detrimental effects on animal health and performance. The impact of glucosinolates remains ambiguous because of inconsistent findings in previous studies. This study analyzed data from 36 research articles to evaluate the influence of glucosinolates on ruminant growth, digestion, milk composition, and methane emissions. These results demonstrated that glucosinolate intake enhanced nutrient digestibility and elevated milk urea nitrogen levels. Furthermore, methane emissions decreased with increased glucosinolate intake. However, the effects varied depending on the source and quantity of glucosinolates, as well as the animal species and dietary composition. These findings elucidate the potential of glucosinolate-containing feeds to enhance ruminant nutrition, mitigate environmental impacts, and underscore the need for careful formulation to optimize benefits and minimize risks. This study contributes to the development of more sustainable livestock feeding strategies that may improve animal productivity and reduce greenhouse gas emissions.

## 1. Introduction

The quest for sustainable and environmentally friendly animal feed solutions has become increasingly imperative because of the global demand for reduced environmental impact and cost-effective livestock production. The traditional reliance on soybean meal as the primary protein source in animal diets has raised economic and environmental concerns, necessitating the exploration of alternative sources of plant proteins. Among these alternatives, *brassica*-derived feeds have garnered significant attention, not only for their competitive pricing but also for their potential as sustainable protein sources in ruminant diets [1]. However, a major challenge with *brassica*-based feeds is the presence of glucosinolates (GLs), which are sulfur-containing secondary metabolites with both nutritional benefits and drawbacks for ruminants. GLs found abundantly within the *Brassica* genus (e.g., *Brassica napus*, *Brassica campestris*, *Brassica juncea*, and *Brassica rapa*) can exert diverse effects on ruminant health and performance.

Although intact GLs generally pose minimal risks, their hydrolysis products, such as nitriles and thiocyanates, have been associated with adverse health outcomes, including hepatorenal enlargement and goiter as well as reduced ammonia and protein digestibility [2,3,4]. These breakdown products not only affect nutrient utilization but also contribute to the bitter and pungent taste of *brassica* feeds, resulting in reduced feed intake and decreased growth rates in ruminants [5].

Regulatory bodies have addressed these challenges through directives aimed at controlling GLs levels in animal feeds. For instance, Directive 2002/32/EC initially restricted the use of *Camelina sativa*, a GL-rich *Brassica* species, in livestock diets; however, subsequent revisions permitted its inclusion at controlled GL levels [6,7]. Despite these regulations, there is no universally accepted threshold for GL intake by ruminants. The suggested upper limits range widely, from 4.4 to 11 µmol/g of diet depending on the animal species and age [8], reflecting the necessity for a more standardized approach to GL management in ruminant nutrition. Although ruminants generally exhibit greater tolerance to GLs than monogastric animals do, prolonged exposure to high GLs levels can have detrimental effects on health and productivity [9] Nevertheless, GLs also have potential benefits, and recent studies suggest that the high GL content in *brassica* forages may function as a hydrogen sink in the rumen, potentially reducing methane emissions, which is an important consideration for sustainable livestock production [10,11].

However, inconsistent findings regarding the effects of GLs on ruminant performance underscore the need for a systematic analysis to establish clear guidelines. Some studies have found no adverse effects on ruminant performance at lower GL intake levels (e.g., below 38.3 µmol/animal/day) [4,12], while others have reported reduced performance at similar or higher levels [13,14,15].

The intricate and often contradictory nature of existing research underscores the critical need for a comprehensive modelling approach to illuminate the relationship between GL consumption and crucial performance indicators in ruminants. In this study, we performed a meta-analysis of in vivo experiments to ascertain the effects of different GL levels on ruminal fermentation, methane production, milk composition, and other biochemical parameters. It has been proposed that meta-analysis may serve as a powerful tool to identify optimal levels of glucosinolate intake, with the potential to improve ruminant performance while reducing associated environmental impacts.

## 2. Materials and Methods

### 2.1. Literature Search and Selection Processes

The database was compiled from the published research articles sourced from Scopus (https://www.elsevier.com/products/scopus, accessed on 30 January 2025), Google Scholar (https://scholar.google.com/, accessed on 30 January 2025), PubMed (https://pubmed.ncbi.nlm.nih.gov/, accessed on 30 January 2025), ScienceDirect (https://www.sciencedirect.com/, accessed on 30 January 2025), and Web of Science (WOS; https://www.webofscience.com, accessed on 30 January 2025) that were systematically selected following Preferred Reporting Items for Systematic Reviews and Meta-Analyses (PRISMA) protocol [16]. The keywords for searching published articles were “glucosinolates”, with “ruminant”, or “rumen”, or “dairy cattle”, or “steers”, or “lambs”, “ewes”, or “yak”, or “kids”, or “sheep”. All included articles compiled in the database were considered generally publisher websites that were indexed in Scopus and WOS.

**Figure 1:**
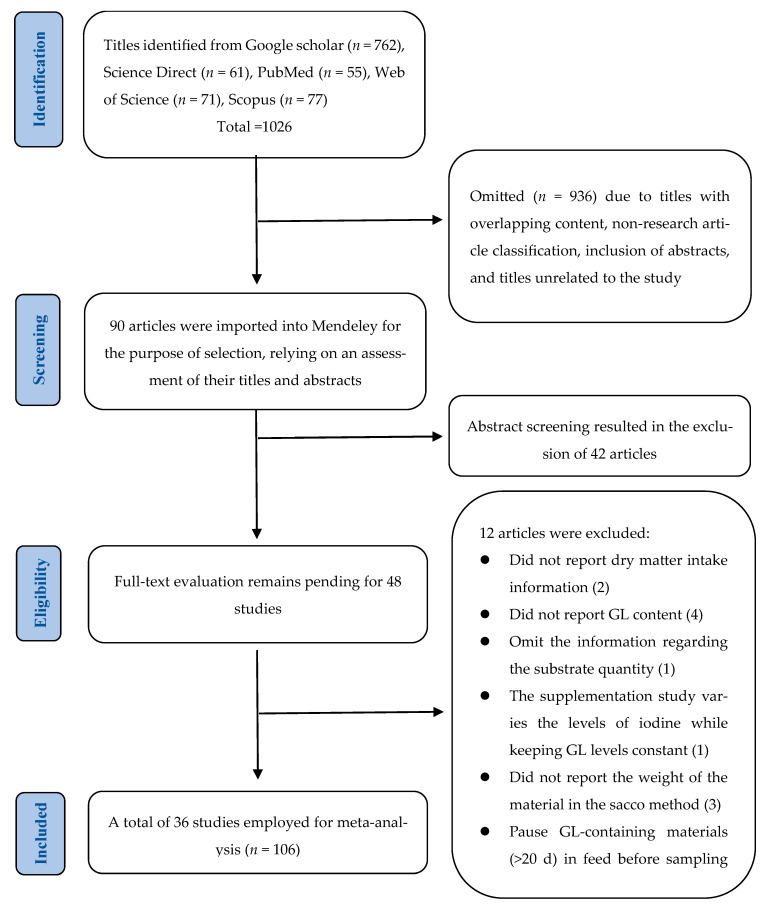
Flowchart of article selection based on the PRISMA protocol.

Moreover, data from the articles were verified using primary selection criteria for inclusion in the database, as follows: (1) publication of original articles in English; (2) conducting in vivo experiments to evaluate the effects of GLs on ruminants at any stage/type; (3) reporting the analytical values and intake of GLs from the diet along with supplement levels or allowing for estimations of dietary GL intake; (4) documenting the effects of GLs on ruminal fermentation, methane emissions, milk composition, or animal biochemical attributes; (5) providing sufficient details on research methodology, including animal randomization, sample size, feed formulations and compositions, data collection and analysis procedures, and statistical methodologies; and (6) confirming approval of the experiment by local ethics organizations. There were no restrictions on publication year. However, articles were excluded if they did not meet specific criteria: (1) failure to report GL levels or dry matter intake; (2) lack of measurement for targeted indicators; (3) absence of reported variances, such as standard deviation or standard error of the mean; and (4) instances where GL-containing materials were withheld from feed for more than 20 days before sampling. Disagreements regarding article inclusion were resolved through discussion by the authors. Moreover, the selection processes are detailed in the PRISMA flowchart based on the double-screen procedure [15] depicted in Figure 1. A total of 36 studies were deemed eligible for the meta-analysis, with the experimental conditions outlined in Table 1 and a quantitative summary provided in Table 2.

### 2.2. Database Development

The database compiled information from selected articles, covering various data points such as animal numbers per treatment, breed, physiological and lactational stages, days in milk (DIM), parity, study duration, experimental design, and statistical methods. It included dietary details like GL levels, dry matter intake, and nutritional composition, alongside response variables such as growth performance (e.g., body weight, daily gain, feed conversion ratio), slaughter data (e.g., carcass weight), and lactation performance (e.g., milk yield, intake metrics, and milk composition). Additionally, it recorded total tract digestibility, nitrogen metabolism, gaseous emissions (e.g., carbon dioxide, methane), biochemical markers, and fermentation traits, along with variances in these parameters.

The standard error of the mean (SEM) was calculated using the formula SE = standard deviation/sqrt (*n*), where n represents the sample size or number of replicates [42]. To enhance data precision and enrich the dataset, additional variables were extracted from graphical data using WebPlotDigitizer (https://apps.automeris.io/wpd/) (accessed on 27 January 2024), maximizing the retrieval of data points. When studies included multiple experiments, each was encoded separately to ensure clear differentiation. The normalized inverse of the SEM, denoted as VAR, was used as the weighting factor for response variables, according to the formula VAR = W1/W2, where W1 represents the reciprocal of the SEM for the specific experiment, and W2 denotes the overall mean of W1 across all experiments, as proposed by Respati et al. [43].

Dietary GL content, primarily originating from *Brassica* genus plants or by-products in concentrate and TMR diets, was incorporated into the database (Table 1). The negligible presence of organic or native GLs in other ingredients rendered their contribution insignificant, as these were either minimal or unspecified in the studies. Consequently, dietary GL data were directly derived from diet compositions. The daily GL intake per animal (expressed in mmol/d/animal) was calculated by multiplying the GL concentration in the diet by the daily dry matter intake (DMI) in kilograms. This approach ensured accurate representation of dietary GLs within the dataset.

### 2.3. Data Analyses

The original database encompassed 302 response variables (*n* = 106 involved treatments), including growth performance, milk production and quality, carcass traits, fatty acid profiles, nutrient intake, digestibility, nitrogen partitioning, gas production, blood biochemistry, rumen fermentation, hormonal and reproductive metrics, enzyme activities, and microbiome composition. After rigorous evaluation, 60 parameters from in vivo studies and six in sacco studies were selected for meta-analysis. Data inspection involved visual assessments using PROC REG in SAS software (v9.4) to examine heterogeneity and exclude outliers, defined by studentized residuals within −3 < t < 3 or Cook’s distance (−1 < t < 1).

The meta-analysis utilized multiple explanatory variables, both categorical (e.g., animal types, GL sources, treatment groups) and continuous (e.g., GL intake in mmol/d), to explore their effects on response variables. Regression models, including multivariate linear and nonlinear (quadratic) fits, were constructed using PROC MIXED in SAS (v9.4), following methodologies from previous meta-analyses [42,43,44]. Initially, all explanatory variables and their interactions were included in a full model. Collinearity was assessed using variance inflation factors (VIFs), with variables having VIF > 10 excluded. A backward elimination approach was applied, removing covariates with *p* > 0.10, while model comparisons were conducted using ANOVA (*p* < 0.05) to identify the optimal model based on Akaike’s information criterion (AIC). Linear models were retained if they showed no significant improvement over nonlinear alternatives, as follow:

∆Υij = β0 + β1Xij + β2Xij2 + (β1 × β3…*n*)Xij × Si + εij, [full model]

∆Υij = β0 + β1Xij + β2Xij2 + (β1 × β3…*n* − 1)Xij × Si + εij, [reduced model]

where ∆Yij = estimated response variable based on jth observation in ith experiment, β0 = the first intercept (fixed effect), β1 = slope of linear regression of continuous predictor (fixed effect), β2 = slope of the quadratic term of continuous predictor (fixed effect), Xij = glucosinolate intake (mmol/d) of jth observation in ith study, X = the matrix of the continuous predictor variable, β3…βn = coefficient of the categorical variables, Si = the random effect of the experiment, and εi = the residual error at ~N(0,σ2). The normalized inversed variance matrix was used in the WEIGHT statement [43]. To do so, the normalized inverse variance was calculated using the SEM of each study as W1/W2, where W1 = 1/SEM of the study and W2 = overall mean of W1 across studies. In cases where the SEM was not reported, the SEM was calculated from the standard deviation (SD) value as SEM = SD/sqrt(n), where *n* is the number of replications. Studies with extremely low SEM values could lead to overweighting. To avoid this, the extremely low SEM value was trimmed and replaced with an adjusted value of 0.35 × overall mean of the SEM [42,43]. In addition, the robustness and accuracy of the model was assessed using the regression plot between observed and predicted values for selected response variables. Comprehensive model evaluation and validation was not performed due to the relatively small sample size.

Furthermore, a categorical mixed model meta-analysis was performed using the categorical treatment groups within studies [42]. The categorical groups were classified as fixed effects, and the individual study was treated as random effects. The following statistical model was used:

Yij = µ + β a + (βa × βb)x ij + sβij + Si + eij

where Yij = the weighted means estimated of the response variable Y of jth observation in ith study, µ = overall mean, βa = fixed effect of categorical data, βb = fixed effect of covariates, βa × βb = interaction terms between categorical data and covariates of jth observation in ith study, sβij = random interaction between i study and the j treatment group of factors β, Si = random effect of the study, and eij = residual error ~N(0,σ2). A significant effect was declared at *p* < 0.05, and a tendency was stated when the *p*-value was between 0.05 and 0.10. Tukey–Kramer’s test was used to test the significance of the least square means of the response estimates.

## 3. Results

### 3.1. Datasets

The literature search in this meta-analysis encompassed 36 published articles that investigated the inclusion of GLs in ruminant diets, along with information such as animal type, breed, production phase, and GL source, in which GL treatments were predominated based on the utilization of rapeseed meal (Table 1). Table 2 presents descriptive data indicating that the range of measured parameter values was within the expected limits, although some response parameters exhibited considerable variability, as evidenced by the large SD values. This variability can be attributed to differences in animal age, production phase, and type of animal measured.

### 3.2. Influences of Increased GL Levels on Ruminant Intake, Digestibility, Performance, and Milk Characteristics

The increased levels of various GLs did not significantly affect ruminant intake, digestibility, and production performance, except for a tendency toward increased carcass weight in a linear manner (*p* = 0.058; Table 3). The increased levels of various GLs also had no significant influence on milk yield and milk nutrient parameters, except for a quadratic response in the increased concentration of milk urea nitrogen (*p* = 0.017).

However, significant results were obtained for ruminant intake where the level of GLs interacted with other variables. These interactions included intake with the type of animal (I × An) for DMI and organic matter intake (OMI) expressed as kg/d (*p* < 0.001), as well as intake with type of GL sources (I × S) and different treatments with the type of animal (TRT × An) for DMI/metabolic body weight (BW), DMI, OMI, neutral detergent fiber intake (NDFI), and acid detergent fiber intake (ADFI) (*p* < 0.05). Regarding crude protein intake (CPI), it tended to exhibit a significant interaction between the levels of GLs and different type of source (I × S; *p* = 0.075). For the digestibility output, increased levels of GLs significantly interacted with different types of animals based on dry matter digestibility (DMD) and crude protein digestibility (CPD) parameters. The significant interaction between increased levels and different types of GLs was also shown for CPD (*p* = 0.026). Moreover, the interaction of TRT × An with all digestibility parameters, such as DMD, organic matter digestibility (OMD), neutral detergent fiber digestibility (NDFD), and CPD (*p* < 0.05), was significant.

Significant interactions were shown for the final BW expressed as kg (*p* = 0.006) and metabolic BW^0.75^ for I × An and TRT × An (*p* < 0.05). Similarly, ruminant average daily gain expressed as ADG g/d and metabolic ADG^0.75^ g/d exhibited significant interactions between type of animal and treatments (I × An; *p* < 0.001), but it showed an a tendency for interaction when expressed as TRT × An (*p* = 0.088). Carcass weight was notably influenced by the interaction between treatments and type of animal (TRT × An, *p* < 0.001), with no other significant effects observed for feed conversion. Moreover, the interaction between increased intake and different sources of GLs (I × S) did not significantly affect most production performance parameters.

Regarding milk production and metabolism characteristics, no significant interactions were found for milk yield parameters expressed as kg/d and milk yield/DMI. However, the interaction between type of GL sources (I × S) influenced milk composition parameters, including 4% fat-corrected milk (FCM) expressed as kg/d, energy-corrected milk (ECM) expressed as kg/d, and proportion of milk protein and lactose (*p* < 0.05). Additionally, milk lactose yield (kg/d) was also significantly influenced by I × S (*p* = 0.014), with milk fat yield showing a trend for a response (*p* = 0.096). On the other hand, the concentration of milk urea nitrogen (MUN), milk true protein kg/d, milk fat proportion and the FCM/DMI (g/kg), did not show significant effects across interaction models.

### 3.3. Influences of Increased GL Levels on Enteric Methane Emission and Rumen Fermentation Characteristics in Ruminants

There were no significant effects on CO_2_ (kg/d) and CH_4_ (g/d) models based on the increased levels of GLs, but the interaction with TRT × An was significant (*p* < 0.01; Table 4). Moreover, increased levels of GL uptake showed a significant reduction in CH_4_ expressed as g/kg DMI in a quadratic manner (*p* = 0.003) with an interaction effect on TRT × An (*p* = 0.029).

For the fermentation characteristics, pH, total volatile fatty acids (VFAs), and valerate were significantly affected by the increased levels of GLs. However, propionate and isobutyrate were both increased in a quadratic and linear manner, respectively, with increased GL uptake (*p* < 0.05), respectively. These changes were further influenced by interactions with I × An (*p* = 0.024) and I × S (*p* = 0.025) on propionate as well as I × An (*p* = 0.013) and TRT × An (*p* = 0.004) on isobutyrate. Meanwhile, acetate increased quadratically (*p* = 0.047), whereas butyrate and the A:P ratio decreased linearly with increased GL uptake (*p* = 0.003). There were with no interaction effects observed for acetate and butyrate, but interactions were noted between I × An (*p* = 0.025) and I × S (*p* = 0.027) on the A:P ratio. There was no effect of increased GL uptake on NH_3_ concentration, except for the interaction effect with I × S (*p* < 0.001).

### 3.4. Influences of Increased GL Levels on Ruminant Nitrogen and Iodine Metabolism as Well as Serum and Blood Plasma Characteristics

Concerning N metabolism in ruminants, parameters such as N intake, N digested, and milk N showed trends toward significance. N intake increased linearly (*p* = 0.061), while N digested (*p* = 0.081) and milk N (*p* = 0.091) decreased linearly with the increased level of GL uptake (Table 5). Meanwhile, other parameters such as fecal N, urinary N, N excretion, and N retention were not affected by the increased levels of GLs. No significant interaction was noted between milk N and N retention. However, the I × An interaction was only significant for N digested (*p* < 0.001), whereas fecal N, N excretion, and N retention significantly interacted with I × S (*p* < 0.05). Urinary N and N intake significantly interacted with TRT × An (*p* < 0.05), whereas N digested, fecal N, and N excretion showed tendencies for interactions (*p* < 0.1).

### 3.5. Predictive Meta-Analytic Models of Digestibility, Rumen Fermentation, Thyroid Serum Parameters

**Figure 2:**
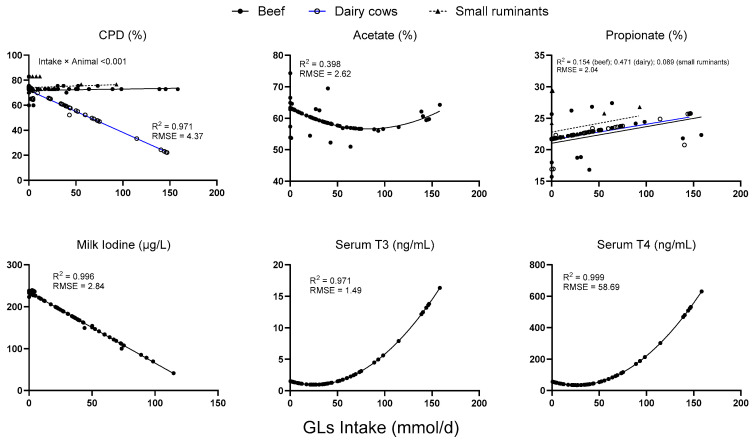
Predicted regression models of several parameters in response to increased glucosinolate intake.

Increased GLs intake marked decline in crude protein disappearance (CPD) in dairy cows (Figure 2), while it remained approximately at baseline levels for beef cattle and small ruminants (intake × animal *p* ≥ 0.001; R^2^ = 0.971). The proportion of acetate initially decreased, followed by a slight rebound at higher intake levels (R^2^ = 0.398). In contrast, propionate exhibited a modest upward trend that was species dependent (beef R^2^ = 0.154; dairy R^2^ = 0.471; small ruminants R^2^ = 0.099). Moreover, the concentration of iodine in milk decreased almost linearly with increased intake (R^2^ = 0.995). Conversely, both serum T_3_ and T_4_ levels remain low at lower intakes and then increase exponentially beyond approximately 50 mmol/d of GLs intake (T_3_: R^2^ = 0.971; T_4_: R^2^ = 0.999), suggesting a threshold-driven thyroid response to elevated glucosinolate exposure. 

**Figure 3:**
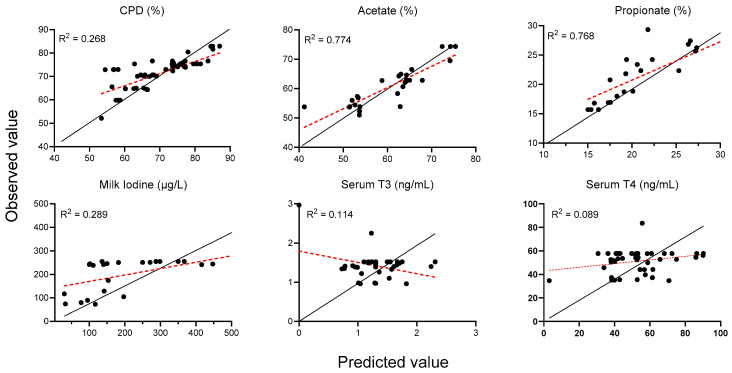
Predicted vs. observed value plots of the meta-analysis of in vivo studies. The red and black solid lines represent the fitted regression line for the relationship between the predicted and observed values and the identity line (y = x), respectively.

In the comparison between the predicted and observed values, acetate (R^2^ = 0.774) and propionate (R^2^ = 0.768) demonstrated a strong linear concordance between the model and empirical data, with the majority of data points closely aligned with the fitted regression (red) and identity (black) lines (Figure 3). The CPD exhibited a moderate fit (R^2^ = 0.268), indicating a greater degree of scatter and tendency for under-prediction at higher values. Similarly, milk iodine displayed a modest correlation (R^2^ = 0.289), with the model generally underestimating at low concentrations and overestimating at high concentrations. Both serum T_3_ (R^2^ = 0.114) and T_4_ (R^2^ = 0.089) exhibited poor predictive performance, as evidenced by the wide dispersion of data points and regression slopes that significantly deviate from the identity line, underscoring the meta-analytic model’s limited capacity to capture the variability of in vivo thyroid hormone levels.

**Figure 4:**
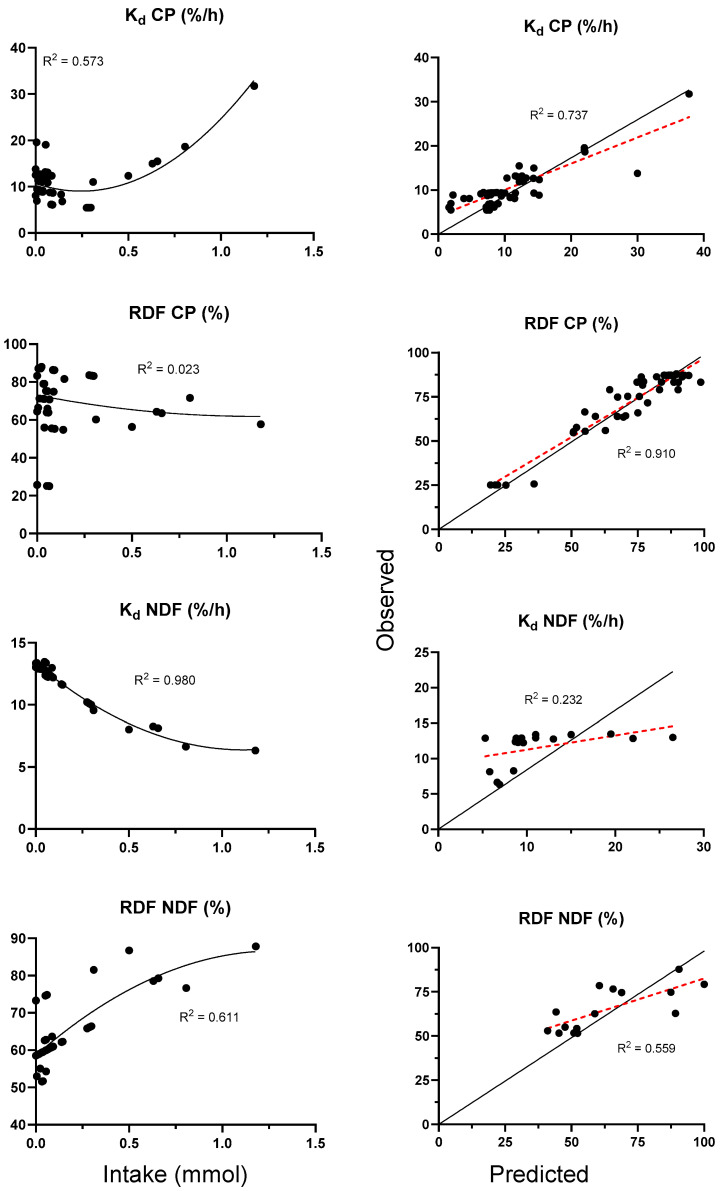
Relationships between levels of glucosinolates and in situ CP and NDF degradability. The red and black solid lines represent the fitted regression line for the relationship between the predicted and observed values and the identity line (y = x), respectively.

Meanwhile for the nutrient degradation (Figure 4), in situ CP degradation rate (Kd CP) exhibited a curvilinear increase (R^2^ = 0.573), with the fitted model predicting the observed values with a reasonable accuracy (predicted vs. observed R^2^ = 0.737). Conversely, the residual degradable CP fraction (RDF CP) remained largely constant across varying intake levels (R^2^ = 0.023) but was predicted with high precision using the prediction equation (predicted vs. observed R^2^ = 0.910). In contrast, the in situ NDF degradation rate (Kd NDF) decreased sharply, following an exponential decay pattern as glucosinolate levels increased (R^2^ = 0.980), although the predictive model demonstrated poor performance (predicted vs. observed R^2^ = 0.232). Additionally, the residual degradable NDF fraction (RDF NDF) exhibited a moderately nonlinear increase with intake (R^2^ = 0.511), accompanied by a modestly accurate prediction fit (predicted vs. observed R^2^ = 0.559).

The iodine content in milk expressed as µg/L milk production and % of total iodine intake decreased linearly (*p* = 0.024) and quadratically (*p* = 0.05), respectively, with increasing levels of GLs. Moreover, regarding serum characteristics, serum T3 and T4 decreased in a quadratic manner (*p* < 0.001), with serum T4 showing a trend for interaction with I × N (*p* = 0.096). Meanwhile, there were no effects of increased GL uptake on blood plasma concentrations of urea-N and glucose as well as albumin, globulin, and blood urea nitrogen (BUN). However, TRT × An interactions were observed for urea-N, glucose, and BUN in blood plasma (*p* < 0.01), whereas total protein showed a trend toward interaction with TRT × An.

### 3.6. Comparative Analysis of Glucosinolate Intake in Different Types of Animals

The comparative effects of glucosinolate inclusion across the different ruminant species are presented in Table 6. The results indicated that the addition of glucosinolates (TRT) to diets had no significant effects on ruminant performance parameters such as final BW, ADG, DM intake, CP intake, and DM digestibility. However, NDF intake, NDF digestibility, and CP digestibility exhibited co-dependence based on the type of animal (TRT × An; *p* < 0.01). Meanwhile, in dairy cows, a tendency for increase milk yield expressed as kg/d was observed (*p* = 0.06), accompanied by an increase in the milk fat proportion (kg/d) in fermented feed containing glucosinolate (FRS), compared to the CON group (*p* = 0.05).

## 4. Discussion

### 4.1. Influences of Increased GL Levels on Ruminant Intake, Digestibility, Performance, and Milk Characteristics

GLs are sulfur-containing phytochemicals predominantly found in cruciferous vegetables, such as broccoli, cabbage, kale, and Brussels sprouts, and are recognized for their anti-inflammatory properties. GLs can be hydrolyzed by the enzyme myrosinase (β-ioglucomides), which is released upon damage to plant tissues (e.g., through mechanical disruption or mastication). This enzymatic reaction converts glucosinolates into various bioactive compounds, including isothiocyanates, nitriles, and indoles, and their mechanism of action can serve as health-promoting agents for humans and animals [45,46]. Numerous studies have systematically investigated the utilization of GLs in ruminant nutrition, and their distinctive properties have been hypothesized to influence rumen fermentation, nutrient digestibility, and overall animal performance and health [10,13,25,26,37].

In the present study, elevated levels of various GLs did not significantly affect overall intake, digestibility, or production performance; however, a tendency for increased carcass weight was observed with higher GL levels. Although DM and OM intake (kg/d) increased with elevated GL levels, increased DM intake relative to metabolic body weight was associated with animal type and GL sources. Palatability issues associated with different GL sources may vary depending on the animal, subsequently affecting their performance [12,25]. Furthermore, the interaction between GLs and other dietary components, such as fiber and protein, can further complicate their effects on feed intake and performance [46]. Similarly, Nkosi et al. [47] reported that the inclusion of GLs from discarded cabbage reduced the DM intake of South African Dorper lambs, which was attributed to both the increased dietary fiber content and its presence. In contrast, Tripathi and As [48] demonstrated that certain levels of GLs improve fiber digestibility and positively enhance VFA production, which are crucial for ruminant energy metabolism. Low and moderate levels of GL inclusion in ruminant diets are beneficial for enhancing rumen fermentation and nutrient digestibility, although GLs are tasteless to small ruminants.

Current evidence also demonstrates that increased GL uptake affects DM and CP digestibility, emphasizing the efficacy of GLs in modulating nutrient fermentation in the rumen [28,40]. Furthermore, the proportion of propionate increased with increasing GL inclusion in small ruminants, beef cattle, and dairy cows. Notably, the hydrolysis of GLs in the rumen can promote a favorable microbial environment that enhances the breakdown of feed components, thereby improving CP digestibility [12,27]. Sun et al. [37] and Nkosi et al. [47] also confirmed that the greater content of readily fermentable carbohydrates and soluble protein content in GL sources are readily degradable in the rumen. Consequently, the presence of GLs can enhance microbial fermentation in the rumen, which is then associated with increased DM and protein digestibility [27,28]. However, the present results indicate that increased levels of GLs had contrary effects in dairy cows, where CP digestibility linearly decreased (Figure 2). Ruminants tolerate higher levels of GLs than monogastric animals. Nevertheless, excessive intake can result in decreased palatability and feed efficiency [45]. Several factors also warrant consideration regarding the effectiveness of GLs in increasing nutrient digestibility, such as animal type, weight, size, and age, which can affect the efficiency of nutrient absorption from feed containing glucosinolates, depending on the attributes of specific types and concentrations of glucosinolates present in the diet [45,48,49]. This suggests that the anti-nutritive effects of GLs are not limited to non-ruminant animals but can also affect ruminants depending on their physiological condition. Increased GL levels in the diet consequently tended to increase ruminant carcass weight. Although increased levels of GLs might not significantly influence performance metrics such as final BW, ADG, and feed conversion, meta-regression of increased levels of GL uptake evidently enhanced carcass weight. Nonetheless, the interaction between animal type and GL intake clearly showed a growth response in specific ruminant types, i.e., beef cattle and small ruminants, that can be associated with the improved CP digestibility by the increased GL levels (Table 3). A previous study also confirmed the positive effects of GLs on growth parameters. For example, Schulmeister et al. [12] found that GLs from *Brassica carinata* improved nutrient digestibility in beef steers and consequently resulted in an increase of 0.5 kg/d in BW gain. Moreover, Du et al. [25] also reported that lambs fed diets with moderate levels of GLs (200–400 g/kg) from *Brassica napus* had greater BW gain and lower FCR than the control.

These findings indicate that moderate levels of GLs from forage rape can positively influence growth performance in ruminants, which is attributed to the nutritional benefits of these GL feed sources, which provide high protein and favorable amino acid content to be readily degraded and synthesized in the ruminal and post-ruminal digestive tracts. Consequently, this intervention improved performance and carcass weight associated with high digestibility related to GL uptake (Figure 2). Studies have also confirmed that sources consisting of readily degraded organic matter, such as rumen-degradable protein (RDP) and non-fiber carbohydrate (NFC), in ruminant diets can alter rumen microbial activity, optimize microbial protein synthesis, and positively affect digestibility, especially in beef cattle and sheep [50,51,52]. Thus, the synchronization of RDP and NFC in GL sources elucidates their positive effects on CP digestibility in beef cattle and small ruminants, but not in dairy cows. The present results demonstrated that the presence of GLs significantly decreased NDFD, particularly in dairy cows, suggesting a relationship between GLs and distinct nutrient digestibility in different ruminants [12]. GLs are antinutritional compounds that can inhibit cellulolytic microbes from synthesizing fiber in the rumen, which further impairs fiber digestibility and nutrient absorption. It can be postulated that differences in digestive physiology among ruminants also result in different growth sensitivities when fed GLs [8,49].

The lack of significant effects on milk yield and nutrient parameters, except for a quadratic increase in MUN emphasizes the distinct role of GLs on nitrogen metabolism. Evidence by Zhao et al. [53] suggests that while GLs may not enhance milk production directly, they could influence metabolic by-products such as MUN, indicating protein utilization efficiency in ruminants. However, Gao et al. [27] stated that the inclusion of *Brassica carinata* (GLs up to 63.96 mmol/animal/d) can potentially enhance milk yield through improved rumen fermentation efficiency and stimulate microbial activity, thereby increasing the availability of energy sources for milk production. The fermentation of GLs can influence the production of propionate, which is particularly important for glucose synthesis and milk lactose production [28,54]. For instance, GLs consist of anti-nutritive compounds such as isothiocyanates (ITCs), thiocyanate (SCN), and goitrin, and these compounds are released when GL source tissues are damaged (e.g., through chopping or chewing). When dairy cow ingest GLs, the myrosinase enzyme is activated to breakdown GL molecules and released ITCs, SCN, and goitrin. These compounds can influence rumen fermentation and nutrient absorption, which are critical for milk production [55,56].

### 4.2. Influences of Increased GL Levels on Enteric Methane Emission and Rumen Fermentation Characteristics in Ruminants

In addition to improved ruminant digestibility of protein, increased GL uptake had beneficial effects on rumen fermentation, as indicated by increased propionate levels and reduced acetate levels. Moreover, the role of bioactive compounds, such as ITCs and SCN, seems not only beneficial for improving rumen microbial activity, but is indirectly followed by a reduction in methane production. Briefly, bioactive compounds are present in major glucosinolate sources; specifically, ITCs, SCN, and goitrin are activated upon ingestion, exhibiting different modes of action in the rumen. The ingested ITCs stimulated the growth of *Fibrobacter succinogenes* and *Ruminococcus albus* in the rumen, facilitating the degradation of fiber in the ingested feed compound to produce VFAs, particularly propionate [12,27].

In contrast, the presence of GLs can lead to the production of secondary metabolites that may inhibit the activity of acetate-producing bacteria, further contributing to the observed reduction in acetate levels [27]. The metabolic pathways that convert carbohydrates into VFAs can be redirected toward propionate production when GLs are present, as these compounds can stimulate the growth of propionate-producing bacteria while suppressing acetate-producing bacteria [4,50]. It is hypothesized that GLs play a role in the SCN by inhibiting methanogenic archaea activities to produce methane; consequently, rumen metabolic pathways are redirected to produce propionate instead of acetate, with methane as by-products [4,10]. This inhibition may result in a shift in microbial community composition, favoring bacteria that produce propionate over those that produce acetate, which can enhance energy efficiency and potentially improve milk yield [4,10].

Propionates also play a role in promoting microbial protein synthesis in the rumen. Increased microbial protein availability contributes to the overall protein supply to lactating cows, which is vital for milk protein synthesis. The balance between propionate and other VFAs such as acetate is crucial for optimizing microbial protein synthesis and overall nutrient utilization [50]. The increased ruminal propionate observed in the present study may have led to increased blood glucose levels, which directly supports lactose synthesis in the mammary gland. However, current meta-analysis findings suggest that increased propionate production, especially influenced by the role of SCN, can potentially explain the enhanced nutrient content in milk, particularly when dairy cows are fed GLs sourced from rapeseed meal [3,4,10,28]. Consequently, it can be postulated that a higher propionate-to-acetate ratio is indirectly associated with increased milk fat and protein content, as propionate is a more energetically favorable substrate for milk synthesis than acetate [57]. Therefore, improved digestibility in ruminants is closely associated with a reduced rumen acetate:propionate ratio when fed with GL sources, even at low and moderate levels.

In the present study, the meta-regression model indicated that in vivo methane production in ruminants was also reduced by increased GL uptake when expressed as CH_4_/DMI. Although the number of observations is limited, several studies corroborate the current evidence regarding the efficacy of GLs in reducing enteric CH_4_ production in ruminants while enhancing production performance.

GLs found in *Brassica* species undergo hydrolysis in the rumen to produce thiocyanate and isothiocyanates, which influence the microbial activity. Gao et al. [27] reported that rapeseed cake, rich in GLs, increased thiocyanate concentrations in the rumen, inhibiting methanogenic archaea and reducing methane emissions. They also demonstrated that steers fed high-GL diets exhibited altered rumen fermentation, leading to lower methane output. Similarly, Sun et al. [37] found that lambs fed fresh winter forage rape emitted less methane than those that consumed perennial ryegrasses, which was attributed to a higher proportion of nutrients that promote propionate production and reduce hydrogen availability for methanogenesis. This evidence substantiates the benefits of GL-modified rumen microbial activity in favoring propionate production, while suppressing methanogenesis.

However, Schulmeister et al. [12] noted that while *Brassica carinata* contains GLs, the methane-mitigating effects can vary depending on the GL concentration. They observed that excessive GL intake may negatively impact growth performance, presenting a trade-off between methane reduction and productivity [12]. Moreover, Zhao et al. [53] further reported that detoxifying GLs with copper sulfate did not significantly improve digestion or methane reduction, suggesting that the effectiveness of GLs may depend on the dietary context and interactions with other anti-nutritional factors.

This implies that the variability in the effects of GLs across studies may be attributed to differences in the GL type and concentration, animal models, and diet composition. Despite the variability in results, it is noteworthy that limited data exist regarding methane-related microbial populations in the context of GL consumption. Nevertheless, the presence of isothiocyanates and thiocyanates derived from GLs consistently demonstrated a reduction in methanogenic activity, substantiating the hypothesis that GLs can effectively mitigate methane emissions in ruminants.

### 4.3. Influences of Increased GL Levels on Ruminant Nitrogen and Iodine Metabolism as Well as Serum and Blood Plasma Characteristics

An investigation into the effects of elevated GL levels on nitrogen and iodine metabolism as well as on serum and blood plasma characteristics in ruminants revealed a complex association with animal physiology. Digested nitrogen was increased; however, the deposition of nitrogen in milk yield was reduced, which was associated with increased GL uptake. This condition reflects a decline in the nitrogen utilization efficiency. Studies have demonstrated that certain GLs can inhibit the growth of beneficial rumen bacteria, leading to reduced protein degradation and absorption [50]. This phenomenon could be attributed to the presence of GLs and their breakdown products, particularly goitrin, which can adversely affect the rumen microbial population responsible for protein fermentation and, subsequently, lower nutrient absorption.

Although similar to the enzymatic activity of myrosinase, unlike isothiocyanates and thiocyanate, the goitrin compound has been confirmed to be associated with goitrogenic effects, which can interfere with iodine metabolism and thyroid function by inhibiting iodide trapping in the thyroid gland. Its mechanism of action inhibits thyroid peroxidase and impairs thyroid hormone synthesis, particularly the production of thyroxine (T4) and triiodothyronine (T3) [15]. For instance, when ruminants are fed GLs with high goitrin content, the formation of the sodium iodide symporter responsible for iodide uptake into thyroid follicular cells is inhibited, further impairing the availability of iodine essential for T4 and T3 hormone synthesis in the thyroid glands. Such iodine insufficiency leads to decreased T4 production, which can have various physiological consequences, including growth retardation and metabolic dysfunction [4].

However, increased GL uptake of more than 50 mmol/d quadratically enhanced T3 and T4 serum levels, indicating the altered endocrine status of ruminants (Figure 2). Notably, increased GL uptake leads to the production of goitrogenic compounds such as thiocyanate (SCN) and goitrin, which can inhibit iodine uptake by the thyroid gland. Initially, it was hypothesized that the physiological status and sex of ruminants might respond differently to the goitrogenic effects of GL intake. Nevertheless, evidence demonstrates that increased GLS levels in diets correlate with fluctuations in serum T3 and T4 concentrations in both male and female cattle [4,53]. However, Schulmeister et al. [12] observed that beef heifers exhibited significant increases in serum T3 and T4 levels when fed diets supplemented with high levels of *Brassica carinata* meal. The hormonal response may differ due to variations in endocrine regulation between sexes, wherein heifers, which are in the growth phase, might exhibit greater sensitivity to GL-induced hormonal changes than steers.

Furthermore, unaltered fecal nitrogen, urinary nitrogen, and nitrogen excretion corroborates the hypothesis of the present study regarding the detrimental effects of GL consumption. Nitrogen utilization was ineffective for ruminant production, as evidenced by the decrease in iodine concentration in milk and total iodine intake with higher GL uptake. The goitrogenic activity of goitrin and SCN (metabolized from goitrin) in GLs decreases T4 production. SCN competes with iodide for uptake by thyroid cells, thereby reducing the accumulation of iodine necessary for thyroid hormone synthesis [4]. Evidence indicates that high GL uptake, such as that contained in rapeseed cake, can lead to significant decreases in serum T4 levels in livestock [27,58]. It can be postulated that impaired ADG is also associated with the goitrogenic effects of GL uptake, which may lead to physiological growth retardation of ruminant performance and metabolic dysfunction, particularly in small ruminants [25,27].

### 4.4. Comparative Analysis of the Intake of Various Types of Glucosinolates in Different Types of Animals

Although the inclusion of GLs in the ruminant diet does not uniformly modify key performance metrics among animal types, the effects are mediated by species-specific physiological and microbial responses. The evidence underscores the necessity of considering individual animal responses and comprehensive dietary interactions when evaluating the effects of GLs in ruminants. Varied GL uptake elicits species-specific responses in ruminants, although it does not consistently alter overall performance parameters, such as FBW, ADG, DM intake, CP intake, or digestibility. It significantly affects the intake and digestibility of neutral detergent fiber and crude protein, varying with animal type [59,60]. Variability in GL metabolism likely underpins these findings. For instance, dairy cows tend to exhibit increased milk yield and milk fat proportions when fed high-protein diets [61,62], such as those containing GLs. This effect may arise from enhanced rumen fermentation, which augments the production of VFAs, a crucial energy source for lactation [47,63]. In support of this, studies have demonstrated that including *Brassica* species in ruminant diets improves both milk yield and quality, as evidenced by dairy cows consuming forage rape (*Brassica napus*) silage outperforming those consuming conventional diets [63,64]. Conversely, excessive GL levels can reduce feed intake and impair growth in certain ruminant species, particularly when GL concentrations exceed tolerance thresholds [65]. These adverse effects highlight the critical need for precise dietary formulations to balance the benefits of GLs with their potential anti-nutritional effects.

The divergent responses among species may be attributed to variations in animal genetics, dietary composition, and the specific GL profile. Interactions between animal type and GL intake indicate that certain breeds exhibit greater resilience than others do [59,60]. Furthermore, the dynamic nature of rumen fermentation, which encompasses fluctuations in microbial community structure, nutrient absorption timing, and pH levels, further complicates the effect of GLs on nutrient digestibility and overall performance. For example, VFA concentrations and absorption rates fluctuate after feeding, thereby modulating the efficacy of GLs [47,63]. Overall, the complex interaction relationship between animal type and dietary GLs suggests the necessity in considering individual type and physiological status to improve the performance and productivity of ruminants.

It is worth noting that a subset of the included studies was conducted under grazing or pasture-based conditions. These systems are inherently more variable due to a range of environmental and management factors, including forage composition, seasonal fluctuations, grazing behavior, and soil nutrient status—all of which can influence the glucosinolate content of forage crops and subsequent animal responses. While efforts were made to account for production system differences in the meta-regression analysis, the limited number of grazing trials and the lack of consistent reporting on pasture-specific variables constrain our ability to fully isolate their effects. Therefore, the results derived from these studies should be interpreted with caution, and future research under controlled grazing conditions is warranted to better understand the dynamics of GL supplementation in pasture-based systems.

## 5. Conclusions

This meta-analysis demonstrated that GL supplementation in ruminant diets yields complex, species-specific effects. Although overall performance parameters, such as body weight, average daily gain, and dry matter intake, remained largely unaffected, significant interactions were observed in nutrient digestibility, particularly for NDF and protein. With respect to ruminant type, dairy cows exhibit a tendency toward enhanced milk yield and fat content, which is potentially attributable to improved rumen fermentation and VFA production. Conversely, excessive GL levels can compromise feed palatability and nutrient utilization, resulting in reduced growth performance in certain species such as small ruminants. Furthermore, the alteration of ruminal microbial dynamics by GL-derived metabolites suggests potential benefits in mitigating enteric methane emissions, highlighting an environmentally favorable aspect of GL inclusion. These findings underscore the need for precise dietary formulations and standardized guidelines to optimize GL intake and balance nutritional benefits against potential anti-nutritional effects. Based on the meta-analysis findings, the recommended concentration of GLs for ruminant uptake is approximately 50 to 100 mmol/d, which is considered low to moderate levels. Future research should elucidate the mechanistic pathways underlying these responses to enable targeted strategies to enhance both productivity and sustainability in ruminant production systems.

## Figures and Tables

**Table 1:** Lists of studies included in the meta-analysis.

No	Study	Study Design	*N*	Animal	Production Phase	GL Source	GL Levels (Animal/d)
1	Ahlin et al., 1994 [17]	RCBD ^1^	222	Dairy cow	Mid-lactation	*Brassica napus oleifera*	0–60 mmol
2	Antaya et al., 2019 [18]	RCBD	60	Dairy cow	Mid-lactation	Diets	881–1597 mg
3	Armstrong et al., 1993 [19]	LSD ^2^	25	Lamb		lamina, petiole	2391.94–7217.29 mg
4	Aronen et al., 1992 [20]	RCBD	48	Cattle	Finishing	*Brassica campestris*	0–73.326 mmol
5	Barry et al., 1983 [21]	RCBD	96	Lamb	Growing	*Brassica oleracea*	0–24.6 mmol
6	Böhme et al., 2005 [22]		60	Dairy cow	Mid-lactation (DIM 211)	Crambe press cake	1–114.7 mmol
7	Moura et al., 2023 [9]	LSD	48	Dairy cow	Mid-lactation (DIM 92–109)	Crambe press cake	0–687.1 mg
8	Moura et al., 2017 [23]	LSD	32	Lamb		Crambe meal	0–114.26 mg
9	Derycke et al., 1999 [24]		66	Lamb	28 ± 8 d	Rapeseed meal	0–3.58278 mmol
10	Du et al., 2022 [25]	CRD ^3^	50	Lamb	Growing	Forage rape	0–5.10534 mmol
11	Durge et al., 2014 [11]	CRD	80	Goat	Early lactation	*Brassica juncea* oil meal	0–11.7576 mmol
12	Fiems et al., 1985 [26]		68	Steer	Growing	Rapeseed meal	0–2577.01 mg
13	Gao et al., 2021 [4]	LSD	32	Cattle	Finishing	Rapeseed meal (heated)	0–63.8577 mmol
14	Gao et al., 2022 [27]	LSD	32	Steer	Finishing	Rapeseed meal	0–63.96 mmol
15	Gao et al., 2023 [28]	CRD	20	Dairy cow	Mid-lactation	Rapeseed meal	4.4–43.2 mmol
16	Gao et al., 2023 [28]	Crossover	8	Dairy cow	Mid-lactation	Rapeseed meal	4.4–43.2 mmol
17	Komprda et al., 2002 [29]	Analogous pairs	23	Cattle		Rapeseed meal (heated)	0–2.975 mmol
18	Laarveld & Christensen, 1976 [30]	LSD	36	Dairy cow		Rapeseed meal	0–15902 mg
19	Lancaster et al., 1990 [14]	LSD	125	Steer		Rapeseed silage	0–158.41 mmol
20	Lancaster et al., 1990 [14]	CRD	60	Steer		Rapeseed silage	0–88.97 mmol
21	Lardy and Kerley, 1994 [31]	RCBD	60	Steer		Rapeseed meal	0–145.54 mmol
22	Papas et al., 1979 [13]		15	Dairy cow	Late lactation	Rapeseed meal	0–73.486 mmol
23	Papas et al., 1979 [13]		9	Dairy cow		Rapeseed meal	0–34.86 mmol
24	Qin et al., 2023 [32]	LSD	64	Dairy cow	Mid-lactation	Rapeseed meal	0–1570.3 mg
25	Ravichandiran et al., 2008 [15]	CRD	18	Steer	Growing		0–38.304 mmol
26	Rezaeipour et al., 2016 [33]	CRD	24	Lamb	Growing	Crambe meal	0–2.985 mmol
27	Schulmeister et al., 2021 [34]	CRD	18	Dairy cow	Heifers	*B. carinata* meal pellets	0–21.01 mmol
28	Schulmeister et al., 2019 [12]	LSD	32	Steer		*B. carinata* meal pellets	0–39.893 mmol
29	Subuh et al., 1995 [35]	LSD	216	Dairy cow		Rapeseed meal	67.2–146.9 mmol
30	Sun et al., 2012 [36]		25	Sheep	Finishing	Kale (*Brassica oleracea* L.)	0.003–1.05 mmol
31	Sun et al., 2015 [37]		84	Lamb	Finishing	Forage rape	0–93.2 mmol
32	Šustala et al., 2003 [38]	LSD	32	Dairy cow		Rapeseed meal	0–22.4 mmol
33	Tripathi et al., 1999 [39]	Switch-over design	20	Sheep		Mustard seed meal	0.912–1.99 mmol
34	Tripathi et al., 2001 [40]		24	Lamb	Growing	Mustard seed meal	0–11140 mg
35	Trøan et al., 2018 [3]	LSD	32	Dairy cow	Mid-lactation (DIM 89)	Rapeseed meal	0–3.19 mmol
36	Veselý et al., 2009 [41]	LSD	8	Dairy cow	Mid-lactation	Rapeseed meal	0–50.14 mmol

^1^: Randomized complete block design; ^2^: Latin square design. ^3^: Completely randomized design.

**Table 2:** Descriptive statistics of recorded data included in the database.

Response Parameters	*n*	Average	Min	Max	SD
*Animal intake*					
DMI/BW^0.75^, kg/d	48	0.1	0.04	0.22	0.04
DMI, kg/d	106	7.98	0.53	23.6	7.56
OMI, kg/d	53	7.44	0.47	21.91	7.08
NDFI, kg/d	60	3.34	0.08	10.27	3.47
ADFI, kg/d	48	1.61	0.07	5.08	1.79
CPI, kg/d	70	1.09	0.01	3.91	1.21
*Digestibility*					
DMD, %	55	67.83	50.9	89	9.19
OMD, %	55	73.32	52.74	91.8	10.39
NDFD, %	52	59.72	39.5	82	11.77
CPD, %	53	70.68	53.4	87	9.17
*Production performance*					
Final BW^0.75^, kg	44	47.78	10.17	135.8	42.82
ADG^0.75^, g/d	51	99.67	14.39	219	63.39
Final BW, kg	44	202.4	22.03	698	224.7
ADG, g/d	51	502.6	35	1320	408.7
Feed conversion	27	7.54	2.66	15.2	3.03
Carcass weight, kg	13	95.78	14.01	231	100.9
*Milk production and metabolism characteristics*					
Milk yield, kg/d	44	20.29	11.6	36.3	5.75
4% FCM, kg/d	25	19.75	12	25.98	4.92
ECM, kg/d	16	23.01	13.3	34.1	6.79
Milk thiocyanate, μg/mL	7	25.32	4.07	47.63	12.74
Milk yield/DMI	33	1.1	0.51	1.57	0.3
4% FCM/DMI	23	1.14	0.77	1.41	0.21
Milk fat, %	38	4.15	3.49	4.72	0.27
Milk protein, %	38	3.38	3.08	3.8	0.2
Milk lactose, %	24	4.72	4.32	5.1	0.22
Milk fat, kg/d	34	0.87	0.49	1.41	0.22
Milk true protein, kg/d	28	0.69	0.4	1.18	0.22
Milk lactose, kg/d	20	0.9	0.53	1.69	0.33
MUN, mg/dL	20	16.47	9.7	25.2	4.96
*Enteric methane emission* ^1^					
CO_2_, kg/d	17	5.26	0.84	11.6	4.63
CH_4_, g/d	17	183.3	11.7	474	180.9
CH_4_, g/kg DMI	17	18.96	13.6	22.9	2.48
*Rumen fermentation characteristics*					
pH	27	6.45	5.84	6.91	0.32
Acetate, %	27	60.78	41.2	75.4	8.25
Propionate, %	27	23.31	15.03	34.9	6.4
Butyrate, %	27	11.78	7.57	17.6	2.57
Isobutyrate, %	16	1.31	0.2	2.1	0.52
Valerate, %	23	1.98	0.59	6.3	1.58
Isovalerate, %	19	1.36	0.1	2	0.51
A:P ratio	27	2.91	1.35	5.03	1.08
Total VFA, mmol	36	93.04	52.02	133.1	22.59
NH_3_, mg/dL	24	14.13	3.87	44.1	12.53
*N metabolism and retention*					
N Intake, g/d	58	157.2	1.43	626.2	181
N digested, g/d	38	94.92	12.77	353.2	105.74
Fecal N, g/d	23	57.73	4.35	168	54.42
Milk N, g/d	38	113.8	62.9	189.5	32.41
Urinary N, g/d	23	48.49	3.19	108	40.35
N excretion, g/d	51	132.7	7.9	338.3	85.49
N retention, g/d	29	97.7	7.58	390.2	128.8
N retention	29	36.66	8.06	77.75	22.43
*Iodine (Iod) metabolism*					
Total Iod intake, mg/d	19	34.08	12.8	95.7	27.24
Milk Iod, µg/L	25	207.4	30	579	135.8
Milk Iod, mg/d	19	4.57	1.19	9.6	2.53
Milk Iod, % Iod intake	19	16.9	6.03	41.45	10.03
*Serum and blood plasma characteristics*					
Serum T3, ng/mL	40	2.98	0	20.25	5.24
Serum T4, ng/mL	48	110.9	3.11	835.8	197.8
Plasma urea-N, mg/dL	13	16.5	10.8	28.5	5.81
Plasma glucose, mg/dL	19	59.44	34.2	67.02	9.2
Total protein, g/dL	16	7.11	5.18	9.69	1.65
Albumin, g/dL	13	2.68	1.64	3.77	0.8
Globulin, g/dL	9	4.63	1.37	6.94	1.86
BUN, mg/dL	15	13.24	4.9	36.27	9.1

*n* = sample size; SD = standard deviation; ADG = average daily gain; BUN = blood urea nitrogen; BW = body weight; FCM = fat-corrected milk; ECM = energy-corrected milk; DMI = dry matter intake; OMI = organic matter intake; CPI = crude protein intake; NDFI = neutral detergent fiber intake; ADFI = acid detergent fiber intake; DMD = dry matter digestibility; OMD = organic matter digestibility; CPD = crude protein digestibility; NDFD = neutral detergent fiber digestibility; Milk I = milk iodine; VFA = volatile fatty acids; NH_3_ = ammonia; serum T3 = triiodothyronine hormone; serum T4 = thyroxine hormone. ^1^ Enteric gas emissions were quantified using two primary techniques: the GreenFeed automated emission monitoring system and open-circuit respiration chambers.

**Table 3:** Meta-regression of the effects of dietary glucosinolates on intake, digestibility, performance, and milk production in ruminants.

Response Parameters	*n*	Model	Model Statistics				Significant Indicators				Significant Interaction			AIC*i*
			Intercept	SE	Slope	SE	*p*-Value	R^2^	RMSE	AIC*m*	I × An	I × S	TRT × An	
*Ruminant intake*														
DMI/BW^0.75^, kg/d	48	L	0.1	0.014	0.0001	0.001	0.855	0.014	0.599	−175	0.485	<0.001	0.005	−146
DMI, kg/d	106	L	8.44	1.58	0.0001	0.001	0.992	0.005	0.384	273	<0.001	<0.001	<0.001	343
OMI, kg/d	53	L	6.05	2.61	0.045	0.083	0.74	0.648	3.273	81	<0.001	<0.001	0.002	24
NDFI, kg/d	60	L	3.8	1.47	0.02	0.06	0.879	0.123	6.155	56	0.984	0.021	<0.001	15
ADFI, kg/d	48	L	2.37	1.23	0.01	0.04	0.87	0.001	3.689	29	-	-	0.024	4
CPI, kg/d	70	L	0.96	0.37	0.002	0.002	0.387	0.001	66.62	−16	0.986	0.075	0.496	38
*Digestibility*														
DMD, %	55	L	66.46	3.28	0.52	0.17	0.944	0.108	5.245	343	0.029	0.183	0.021	278
OMD, %	55	L	73.54	3.29	0.91	0.379	0.548	0.004	54.43	417	0.508	0.687	<0.001	272
NDFD, %	52	L	60.14	3.67	−0.42	0.21	0.131	0.006	22.05	350	0.632	0.717	0.006	299
CPD, %	53	L	72.13	2.91	0.0001	0.003	0.989	0.508	4.369	327	0.003	0.026	0.002	185
*Production performance*														
Final BW^0.75^, kg	44	L	49.42	13.13	0.189	0.095	0.588	<0.001	22.87	210	0.006	0.382	0.002	374
ADG^0.75,^ g/d	51	L	98.82	17.03	−0.278	0.427	0.389	0.015	34.59	437	0.006	0.374	0.088	353
Final BW, kg	44	L	207.9	69.35	0.919	0.397	0.687	0.062	121.3	320	0.005	0.817	0.009	361
ADG, g/d	51	L	509.6	158.03	−1.7	2.43	0.533	0.024	144.1	604	0.008	0.148	<0.001	545
Feed conversion	27	L	7.89	1.316	−0.001	0.008	0.862	0.029	0.602	140	0.972	0.601	0.473	151
Carcass weight, kg	13	L	4.9	10.29	19.23	8.72	0.058	0.991	0.004	120	0.407	-	<0.001	103
*Milk production and metabolism characteristics*														
Milk yield, kg/d	44	L	21.76	1.99	0.0004	0.007	0.961	0.098	3.087	245	-	0.937	-	193
4% FCM, kg/d	25	L	20.66	2.13	−0.003	0.008	0.702	0.001	2.035	118	-	0.05	-	93
ECM, kg/d	16	L	25.26	3.95	0.007	0.032	0.834	0.075	2.34	74	-	0.009	-	53
Milk thiocyanate, μg/mL	7	L	17.89	0.97	1.36	1.28	0.474	0.993	0.001	11	-	-	-	9.5
Milk yield/DMI, g/kg	33	L	1.15	0.116	−0.0003	0.0002	0.114	0.07	0.143	−56	-	0.471	-	−35
4% FCM/DMI	23	L	1.17	0.097	−0.003	0.002	0.119	0.054	0.086	−37	-	0.199	-	−35
Milk fat, %	38	L	4.06	0.085	0.006	0.009	0.248	0.187	0.102	25	-	0.567	-	15
Milk protein, %	38	L	3.36	0.07	−0.0001	0.007	0.202	0.001	0.077	7.4	-	<0.001	-	−24
Milk lactose, %	24	L	4.72	0.119	−0.0002	0.019	0.923	0.02	0.087	−10	-	<0.001	-	−32
Milk fat, kg/d	34	L	0.91	0.079	0.002	0.004	0.834	0.007	0.106	25	-	0.096	-	−16
Milk true protein, kg/d	28	L	0.75	0.086	0.002	0.004	0.588	0.001	0.098	−47	-	0.256	-	−43
Milk lactose, kg/d	20	L	1.01	0.19	−0.004	0.005	0.705	0.006	0.127	−26	-	0.014	-	−29
MUN, mg/dL	20	Q	18.09	2.82	−0.08	0.03	0.017	0.593	1.941	86	-	0.598	-	48
					0.0001	0.0001								

*n* = sample size; SE = standard error; R^2^ = determination coefficient; RMSE = root mean square error; L = linear pattern; Q = quadratic pattern; ADG = average daily gain; BW = body weight; FCM = fat-corrected milk; ECM = energy-corrected milk; DMI = dry matter intake; OMI = organic matter intake; CPI = crude protein intake; NDFI = neutral detergent fiber intake; ADF = acid detergent fiber intake; DMD = dry matter digestibility; OMD = organic matter digestibility; CPD = crude protein digestibility; NDFD = neutral detergent fiber digestibility; I = intake; An = animal type; S = glucosinolate source; TRT = the treatment effects of glucosinolates in the diet; I × An = interaction effect between glucosinolate intake and type of animal; I × S = interaction effect between glucosinolate intake and their sources; TRT × animal = interaction effect between glucosinolate treatment and type of animal; AIC*m* = model’s Akaike information of criteria, AIC*i* = interaction model’s Akaike information of criteria.

**Table 4:** Meta-regression of the effects of dietary glucosinolates on gas production and rumen fermentation characteristics in ruminants.

Response Parameters	*n*	Model	Model Statistics				Significant Indicators				Significant Interaction			AIC*i*
			Intercept	SE	Slope	SE	*p*-Value	R^2^	RMSE	AIC*m*	I × An	I × S	TRT × An	
*Gas production*														
CO_2_, kg/d	17	L	5.67	2.75	−0.01	0.03	0.578	0.001	1.581	63	0.133	0.742	<0.001	16
CH_4_, g/d	17	L	195.8	103.8	−0.84	0.77	0.292	0.004	60.75	170	0.265	0.607	<0.001	49
CH_4_, g/kg DMI	17	Q	20.83	0.672	−0.25	0.053	0.003	0.054	4.041	91	-	0.396	0.029	108
					0.002	0.001								
*Rumen fermentation characteristics*														
pH	27	L	6.41	0.15	0.01	0.008	0.115	0.113	0.129	17	0.878	0.342	0.34	26
Acetate (A), %	27	Q	64.87	3.17	−0.196	0.069	0.047	0.427	2.623	160	0.131	0.131	0.19	140
					0.001	0.001								
Propionate (P), %	27	Q	20.31	2.46	0.133	0.05	0.047	0.269	2.039	150	0.024	0.025	0.121	123
					−0.001	0.003								
Butyrate, %	27	L	11.14	1.12	0.15	0.06	0.034	0.26	0.844	116	0.804	0.727	0.345	111
Isobutyrate, %	16	L	1.12	0.32	0.02	0.02	0.539	0.196	0.127	36.3	0.013	0.297	0.004	32
Valerate, %	23	L	1.31	0.3	−0.0002	0.03	0.993	0.138	0.135	67	0.452	0.182	0.763	45
Isovalerate, %	19	L	1.13	0.19	0.05	0.03	0.09	0.572	0.078	43	0.023	0.29	0.099	43
A:P ratio	27	L	3.54	0.48	−0.09	0.03	0.003	0.697	0.389	87	0.025	0.027	0.665	77
Total VFA, mmol	36	L	98.55	6.98	0.25	0.75	0.623	0.055	4.681	240	0.503	0.496	0.62	211
NH_3_, mg/dL	24	L	15.04	5.79	−0.14	0.13	0.325	0.1	4.643	124	-	<0.001	0.533	54

*n* = sample size; SE = standard error; R^2^ = determination coefficient; RMSE = root mean square error; L = linear pattern; Q = quadratic pattern; CO_2_ = carbon dioxide, CH_4_ = methane production VFA = volatile fatty acids; NH_3_ = ammonia; I = intake; An = animal type; S = glucosinolate source; TRT = the treatment effects of glucosinolates in the diet; I × An = interaction effect between glucosinolate intake and type of animal; I × S = interaction effect between glucosinolate intake and their sources; TRT × animal = interaction effect between glucosinolate treatment and type of animal; AIC*m* = model’s Akaike information of criteria, AIC*i* = interaction model’s Akaike information of criteria.

**Table 5:** Meta-regression of the effects of dietary glucosinolates on N and Iodine metabolisms, as well as the serum and blood plasma characteristics in ruminant.

Response Parameters	*n*	Model	Model Statistics				Significant Indicators				Significant Interaction			AIC*i*
			Intercept	SE	Slope	SE	*p*-Value	R^2^	RMSE	AIC*m*	I × An	I × S	TRT × An	
*N metabolism and retention*														
N intake, g/d	58	L	124.3	47.97	0.11	0.33	0.061	0.007	66.62	262	0.982	0.854	<0.001	174
N digested, g/d	38	L	96.05	37.29	−0.33	0.52	0.081	0.004	54.43	304	<0.001	0.482	0.056	191
Fecal N, g/d	23	L	44.6	21.44	−0.11	0.79	0.969	0.006	22.06	187	0.898	0.019	0.051	99
Milk N, g/d	38	L	119	12.33	−0.11	0.25	0.091	0.001	15.93	159	-	0.367	0.623	192
Urinary N, g/d	23	L	39.87	17.16	−0.19	0.33	0.572	0.024	16.34	159	0.831	0.44	<0.001	99
N excretion, g/d	51	L	121.4	23.4	−0.14	0.24	0.685	0.014	50.83	389	0.704	0.038	0.054	293
N retention, g/d	29	L	71.5	46.69	0.48	0.86	0.602	0.016	57.69	271	0.419	<0.001	0.79	195
N retention	29	L	35.02	8.32	−0.04	0.25	0.757	0.02	9.962	196	0.909	0.362	0.833	132
*Iodine (Iod) metabolism*														
Total Iod intake, mg/d	19	L	39.2	12.11	−0.91	8.19	0.859	0.977	2.458	146	-	0.636	-	216
Milk Iod, µg/L	25	L	255.1	29.39	−4.12	1.67	0.024	0.997	2.841	295	-	0.281	-	75
Milk Iod, mg/d	19	L	4.74	0.76	−0.24	0.23	0.332	0.999	0.02	99	-	0.281	-	75
Milk Iod, % Iod intake	19	Q	20.61	4.37	−0.51	0.205	0.05	0.436	11.76	129	-	0.563	-	94
					0.007	0.03								
*Serum and blood plasma characteristics*														
Serum T3, ng/mL	40	Q	1.52	0.241	−0.04	0.02	0.008	0.671	1.493	172	0.203	0.905	-	151
					0.001	0.003								
Serum T4, ng/mL	48	Q	57.79	9.86	−1.78	0.824	0.004	0.661	58.69	548	0.096	0.475	-	485
					0.034	0.011								
Plasma urea-N, mg/dL	13	L	18.21	5.15	−0.08	0.53	0.234	0.875	1.956	48	-	0.614	0.022	30
Plasma glucose, mg/dL	19	L	56.59	5.67	−0.012	0.31	0.639	0.009	3.349	112	-	-	0.003	71
Total protein, g/dL	16	L	7.09	0.75	−0.002	0.04	0.256	0.093	0.553	44	0.396	-	0.069	34
Albumin, g/dL	13	L	2.78	0.41	−0.02	0.04	0.562	0.246	0.213	36	0.904	-	0.269	28
Globulin, g/dL	9	L	3.58	2.22	0.27	0.32	0.58	0.025	0.486	26	-	-	0.519	14
BUN, mg/dL	15	L	13.42	4.82	−0.21	0.39	0.668	0.046	2.655	95	0.929	-	0.002	78

*n* = sample size; SE = standard error; R^2^ = determination coefficient; RMSE = root mean square error; L = linear pattern; Q = quadratic pattern; N = nitrogen; Iod = iodine; serum T3 = triiodothyronine hormone; serum T4 = thyroxine hormone; BUN = blood urea nitrogen; I = intake; An = animal type; S = glucosinolate source; TRT = the treatment effects of glucosinolates in the diet; I × An = interaction effect between glucosinolate intake and type of animal; I × S = interaction effect between glucosinolate intake and their sources; TRT × animal = interaction effect between glucosinolate treatment and type of animal; AIC*m* = model’s Akaike information of criteria, AIC*i* = interaction model’s Akaike information of criteria.

**Table 6:** Meta-analysis of the observed variables based on treatment and type of ruminant.

Items	Beef Cattle		Dairy Cows			Small Ruminants		SEM	*p*-Value	
	CON	GLU	CON	GLU	FRS	CON	GLU		TRT	TRT × An
Final BW, kg	228.3	227.1	477.1	477.7	-	33.78	34.98	81.19	0.953	0.836
ADG, g/d	754.3	769.6	414.2	323.9	-	168.9	173	200.7	0.614	0.332
DMI, kg/d	5.22	5.21	18.7	19.1	19.3	1.07	1.02	0.924	0.399	0.173
NDFI, kg/d	2.33 ^c^	2.34 ^c^	7.76 ^b^	8.43 ^a^	8.22 ^a^	0.40 ^d^	0.44 ^d^	1.32	0.002	0.003
CPI, kg/d	0.69	0.68	2.86	2.93	2.82	0.186	0.189	0.345	0.147	0.266
DMD, %	64.2	64.9	66.3	68.6	64.6	71.8	76.1	8.38	0.851	0.379
NDFD, %	46.9 ^d^	46.6 ^d^	57.2 ^bc^	61.7 ^b^	56.1 ^c^	76.4 ^a^	65.7 ^b^	6.59	0.229	<0.001
CPD, %	71.0 ^b^	71.4 ^b^	68.2 ^bc^	65.3 ^bc^	58.1 ^c^	69.4 ^b^	79.2 ^a^	9.78	0.506	0.006
Milk yield, kg/d	-	-	21.6 ^b^	21.8 ^b^	23.4 ^a^	-	-	2.03	0.06	-
Milk yield/DMI	-	-	1.14	1.14	1.17	-	-	0.115	0.662	-
Milk fat, %	-	-	4.03	4.1	4.36	-	-	0.203	0.186	-
Milk protein, %	-	-	3.34	3.37	3.31	-	-	0.109	0.669	-
Milk lactose, %	-	-	4.75	4.7	4.68	-	-	0.129	0.252	-
Milk fat, kg/d	-	-	0.89 ^b^	0.91 ^b^	1.09 ^a^	-	-	0.073	0.05	-
Milk protein, kg/d	-	-	0.74	0.75	0.74	-	-	0.085	0.606	-
Milk lactose, kg/d	-	-	1.01	1	1.01	-	-	0.194	0.892	-

^a–d^ Means in the same row with different superscripts differ (*p* < 0.05); ADG = average daily gain; BW = body weight; DMI = dry matter intake; OMI = organic matter intake; CPI = crude protein intake; NDFI = neutral detergent fiber intake; ADF = acid detergent fiber intake; DMD = dry matter digestibility; CPD = crude protein digestibility; NDFD = neutral detergent fiber digestibility; CON = control group; GLU = glucosinolate effects in the diet; FRS = fermented feed containing glucosinolates; SEM = standard errors of the means; TRT × Animal = interaction effect between glucosinolate treatment and type of animal.

## Data Availability

The dataset underlying the reported results is available from the corresponding authors and will be provided upon reasonable request.

## Abbreviations

The following abbreviations are used in this manuscript:

ADFI	Acid detergent fiber intake
ADG	Average daily gain
AIC	Akaike’s information criterion
AIC*m*	Model’s Akaike information of criteria
AIC*i*	Interaction model’s Akaike information of criteria
BUN	Blood urea nitrogen
CPD	Crude protein digestibility
CRD	Completely randomized design
DMD	Dry matter digestibility
DMI	Dry matter intake
DIM	Days in milk
ECM	Energy-corrected milk
FCM	Fat-corrected milk
GLs	Glucosinolates
ITCs	Isothiocyanates
LSD	Latin square design
MUN	Milk urea nitrogen
NDFD	Neutral detergent fiber digestibility
NDFI	Neutral detergent fiber intake
NFC	Non-fiber carbohydrate
OMD	Organic matter digestibility
OMI	Organic matter intake
PRISMA	Preferred Reporting Items for Systematic Reviews and Meta-Analyses
RCBD	Randomized complete block design
RDP	Rumen-degradable protein
RMSE	Root mean square error
SCN	Thiocyanate
SD	Standard deviation
SEM	Standard error of the mean
VFA	Volatile fatty acid
VIF	Variance inflation factors
